# *Plants* 2022 Best Paper Award

**DOI:** 10.3390/plants11162176

**Published:** 2022-08-22

**Authors:** Plants Editorial Office

**Affiliations:** MDPI, St. Alban-Anlage 66, 4052 Basel, Switzerland; plants@mdpi.com

*Plants* is instituting the Best Paper Awards to recognize the outstanding papers published in the journal. As the editorial office of *Plants*, we are pleased to announce the winners of the *2022 Plants* Year Best Paper Awards.

Papers published in 2020 were preselected by the *Plants* Editorial Office based on the number of citations and downloads from the website. The winner nominations were made by a selection committee, which was chaired by the Editor-in-Chief, Prof. Dr. Dilantha Fernando and supported by seventeen Academic Editors. The four top-voted papers, in no particular order, have won the 2022 *Plants* Best Paper Award:

## 1. Review Paper Award

**Photosynthetic Metabolism under Stressful Growth Conditions as a Bases for Crop Breeding and Yield Improvement [1]**

Fermín Morales, María Ancín, Dorra Fakhet, Jon González-Torralba, Angie L. Gámez, Amaia Seminario, David Soba, Sinda Ben Mariem, Miguel Garriga and Iker Aranjuelo

*Plants* 2020, 9(1), 88; https://doi.org/10.3390/plants9010088

Available online: https://www.mdpi.com/2223-7747/9/1/88

**Figure 1:**
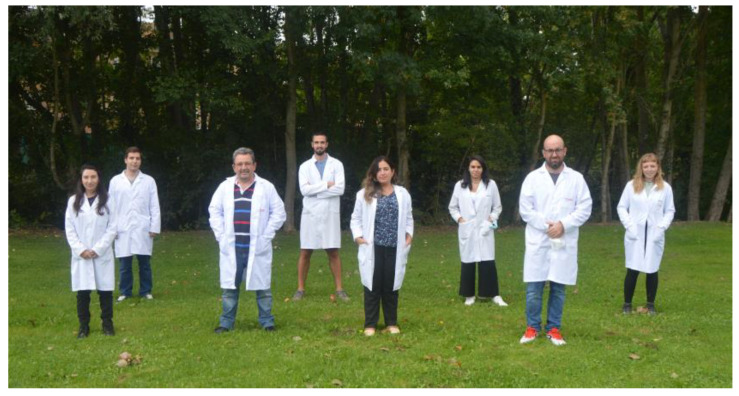
Research group of “Sustainable Agriculture and Biomonitoring” at the Agrobiotechnology Institute (IDAB, CSIC—Gobierno de Navarra) in Mutilva, Navarra, Spain.

This review paper is the result of our experience in science areas such as photosynthesis, plant metabolism and crop stress physiology, in addition to how they influence crop production and their possible applications for crop breeding (Figure 1).

Soil nutrient availability together with increased periods of water shortage and higher temperatures are major factors that negatively impact crops yield. Photosynthetic CO_2_ assimilation (C fixation) is the basis of crop production for animal and human food. For this reason, it has been selected as a primary target for crop phenotyping and breeding strategies. Within this context, knowledge of the mechanisms involved in the response and acclimation of photosynthetic C fixation to multiple changing environmental conditions (nutrients, water, temperature, etc.) is a matter of great concern for the understanding of crops’ behavior under stress, and for the development of tools for enhancing crop production in the future.

The current review analyzes, from a multi-perspective approach (ranging across breeding, gas exchange, genomics and other science areas) the impact of changing environmental conditions on the performance of the photosynthetic apparatus and, as a consequence, on plant growth.

## 2. Research Article Awards

**Plant Roots Release Small Extracellular Vesicles with Antifungal Activity [2]**

**Figure 2:**
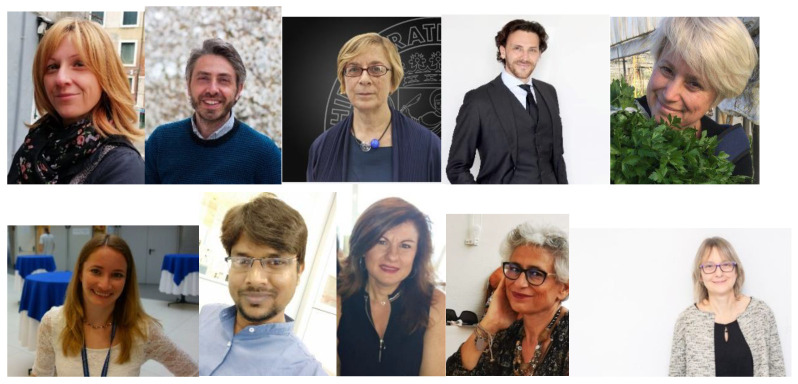
Monica De Palma, Alfredo Ambrosone, Antonietta Leone, Pasquale Del Gaudio, Michelina Ruocco, Lilla Turiák, Ramesh Bokka, Immacolata Fiume, Marina Tucci and Gabriella Pocsfalvi (From left to right, top to bottom).

Monica De Palma, Alfredo Ambrosone, Antonietta Leone, Pasquale Del Gaudio, Michelina Ruocco, Lilla Turiák, Ramesh Bokka, Immacolata Fiume, Marina Tucci and Gabriella Pocsfalvi (Figure 2)

*Plants* 2020, 9(12), 1777; https://doi.org/10.3390/plants9121777

Available online: https://www.mdpi.com/2223-7747/9/12/1777

The paper “Plant roots release small extracellular vesicles with antifungal activity” can be considered as a hallmark in the field of plant extracellular vesicles (EVs). For the first time, we showed that plant cells secrete membrane-enclosed nanometer-sized vesicles outside the cell wall. Our multidisciplinary team demonstrated the physical, molecular and bioactivity features of tomato root-derived EVs using a hydroponics-based experimental setup for the collection of water sampling solution containing the root exudate. Importantly, study of the effect of plant root EVs on the spore germination of different fungal pathogens and shot gun proteomics pointed out the role of plant EVs in defense mechanisms and interspecies communication. The output of this work aided the establishment of the baseline for two Horizon 2020 projects on plant derived EVs (greenEV, GA No. 895579 and nanoTOM, GA No. 798576).

**Feeding Behavior and Virus-transmission Ability of Insect Vectors Exposed to Systemic Insecticides [3]**

**Figure 3:**
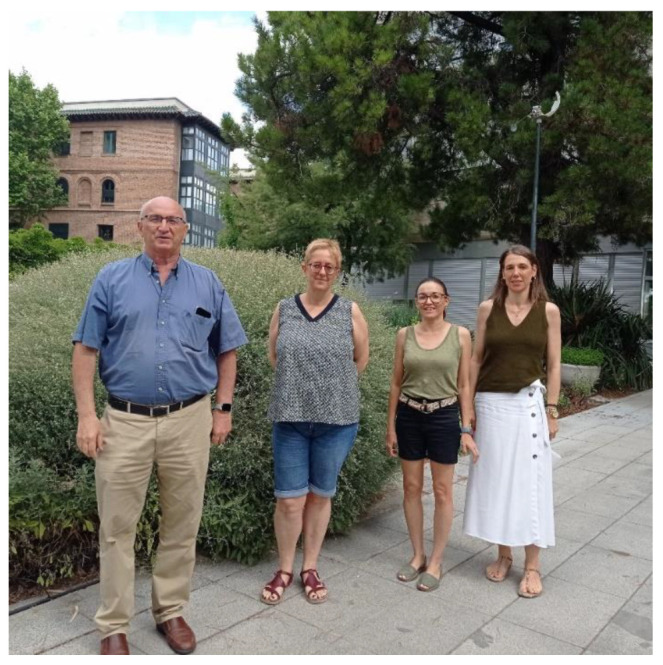
A photo of authors of the article “Feeding Behavior and Virus-transmission Ability of Insect Vectors Exposed to Systemic Insecticides”.

Elisa Garzo, Aránzazu Moreno, María Plaza and Alberto Fereres (Figure 3)

*Plants* 2020, 9(7), 895; https://doi.org/10.3390/plants9070895

Available online: https://www.mdpi.com/2223-7747/9/7/895

The effective management of insect vectors of plant viruses is essential for minimizing vector-borne diseases in crops. In this way, specific chemical compounds may alter the feeding behavior of vectors in a way that transmission of phloem-restricted viruses and plant pathogenic bacteria can be disrupted. However, the insecticides need to act fast enough to prevent long access periods into the phloem/xylem in order to induce feeding cessation. The article report the effect of various systemic insecticides on the feeding behavior of *Bemisia tabaci* and *Myzus persicae*, as well as their ability to interfere with the transmission of two circulative viruses as tomato yellow leaf curl virus (TYLCV) and turnip yellows virus (TuYV). For this study, we used the electrical-penetration-graph (EPG) technique that is a useful tool to understand the transmission mechanisms of plant pathogens by their insect vectors and the mode of action of insecticides. The results show that specific systemic insecticides can be useful for reducing the number of insect vectors. In addition, the antifeeding and feeding cessation effects produced by this systemic insecticides could play an important role in reducing the acquisition and inoculation rate of those viruses transmitted in a circulative persistent manner.

**Transcriptome Analyses and Antioxidant Activity Profiling Reveal the Role of a Lignin-Derived Biostimulant Seed Treatment in Enhancing Heat Stress Tolerance in Soybean [4]**

**Figure 4:**
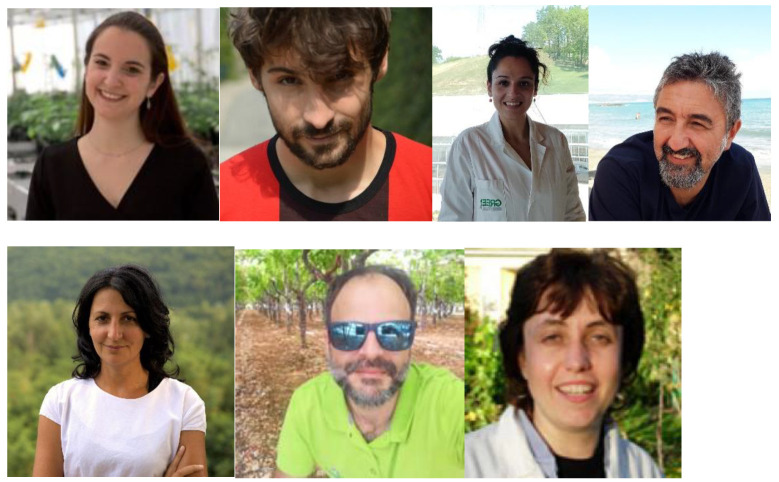
Cristina Campobenedetto, Giuseppe Mannino, Chiara Agliassa, Alberto Acquadro, Valeria Contartese, Christian Garabello and Cinzia Margherita Bertea (From left to right, top to bottom).

Cristina Campobenedetto, Giuseppe Mannino, Chiara Agliassa, Alberto Acquadro, Valeria Contartese, Christian Garabello and Cinzia Margherita Bertea (Figure 4)

*Plants* 2020, 9(10), 1308; https://doi.org/10.3390/plants9101308

Available online: https://www.mdpi.com/2223-7747/9/10/1308

Biostimulants represent a useful and environmentally friendly tool to counteract abiotic stresses. This paper is focused on the study of the role of a biostimulant based on lignin derivatives, plant-derived amino acids, and molybdenum in enhancing soybean (*Glycine max* Merr.) seed germination under high temperature conditions when applied to the seeds prior sowing. Soybean is one of the most largely cultivated crops all over the world and its production is localized especially in countries like Brazil and Argentina, in which high temperatures are often present at sowing time. Hence, the need to find a tool to protect seeds from heat stress and promote the germination under such adverse conditions is of paramount importance. To investigate the biostimulant effect when used as a seed treatment agent and its potential mode of action, a multidisciplinary approach including morphological, biochemical and transcriptional analyses (RNA-Seq) was used. After treatment with the biostimulant in heat stress conditions, the seed biometric parameters were positively influenced and the germination percentage result increased compared to untreated seeds incubated in the same conditions. Moreover, the treatment led to a decrease of oxidative stress and induced the expression of genes, in particular methyltransferases, involved in different processes such as DNA repair, molecule biosynthesis, protection from enzymatic degradation and response to abiotic stresses. In conclusion, the results of this study provide insights on the biostimulant mechanism of action and on its application for seed treatments to improve heat stress tolerance during germination and open a perspective for the use of this product in other crops.

These four outstanding papers are highly valuable contributions to *Plants*. On behalf of the *Plants* Editorial Office, we would like to congratulate these four teams for their excellent work. In recognition of their accomplishments, each team will receive a certificate and a cash award of 500 CHF plus a waiver to enable them to publish a paper free of charge in 2022.

We would like to take this opportunity to thank all the nominated research groups of the above exceptional papers for their contributions to *Plants*, and thank the selection committee for voting and helping with this “Best Paper Award”.

The Editorial Board and Editorial Staffs at *Plants* are committed to meeting the needs of our research community by providing constructive and timely reviews of all quality manuscripts submitted and providing an open access journal for the broad dissemination of your findings. Please consider submitting your work to *Plants*, and we look forward to considering your paper as a *Plants* Best Paper in the future.

**Plants 2022 Best Paper Award Committee,**

Plants Editorial Board.
